# Uterus Cryopreservation From Experimental Models to Clinical Application: A Translational Review

**DOI:** 10.7759/cureus.100012

**Published:** 2025-12-24

**Authors:** Jesus Alberto Sanson-Riofrio, Soledad Ruiz-Matus, Patricia Goldstein, Alvar J Vacio Olguin, Roberto D Robles, Maria del Rosario Garcia, Angelica Morelia, Emmanuel Muñoz Cazola, Manuel M Meraz

**Affiliations:** 1 Surgical Oncology, General Hospital of Zone No. 33 (Bahía de Banderas) Mexican Social Security Institute (IMSS), Puerto Vallarta, MEX; 2 Physics, National Autonomous University of Mexico (UNAM), Mexico City, MEX; 3 Surgical Oncology, Oncology Hospital, National Medical Center Siglo XXI, Mexican Social Security Institute (IMSS), Mexico City, MEX; 4 Surgical Oncology, Mexican Social Security Institute (IMSS), Mexico City, MEX; 5 Surgery, General Hospital of Zone No. 33 (Bahía de Banderas) Mexican Social Security Institute (IMSS), Puerto Vallarta, MEX; 6 Emergency Medicine, General Hospital of Zone No. 33 (Bahía de Banderas) Mexican Social Security Institute (IMSS), Puerto Vallarta, MEX; 7 Epidemiology, General Hospital of Zone No. 33 (Bahía de Banderas) Mexican Social Security Institute (IMSS), Puerto Vallarta, MEX; 8 Clinical Laboratory Medicine, General Hospital of Zone No. 33 (Bahía de Banderas) Mexican Social Security Institute (IMSS), Puerto Vallarta, MEX; 9 Graduate Studies and Research Section (SEPI), National Polytechnic Institute, School of Medicine (ESM, IPN), Mexico City, MEX

**Keywords:** cryopreservation, experimental model, freezing techniques, thawing techniques, uterus

## Abstract

This narrative review provides a preclinical, hypothesis-generating overview of uterine cryopreservation using animal models, with particular emphasis on large-animal experimental systems. Cryopreservation of solid organs remains one of the most significant challenges in preclinical and translational research, driven by persistent organ shortages, limited ischemia tolerance, and logistical and immunological barriers inherent to current transplantation paradigms. Despite decades of progress in cryobiology, no solid organ has yet been successfully cryopreserved, transplanted, and shown to sustain long-term functional outcomes in humans. In this context, the porcine uterus has gained attention as an experimental model for whole-organ preservation because of its structural and functional complexity combined with its non-vital status, offering an ethically acceptable and physiologically relevant platform for methodological development. This narrative review, informed by a structured literature search, examines experimental studies of uterine cryopreservation in animal models. A comprehensive search identified 43 relevant publications, of which 29 were included based on predefined relevance criteria and qualitative appraisal of methodological reporting quality. The synthesis focuses on cryoprotectant formulations, slow-freezing and vitrification strategies, and investigational rewarming approaches, including nanowarming, with particular attention to the porcine uterus as a preclinical model closely approximating human uterine anatomy and physiology. Available evidence indicates that controlled slow-freezing protocols using permeable cryoprotectants can preserve uterine histological architecture and retain partial post-thaw myometrial contractility under experimental conditions. Functional data derive predominantly from small-animal models and limited porcine uterine studies, while robust organ-level comparisons between slow freezing and vitrification remain unavailable. Although advanced rewarming technologies have demonstrated improved thermal uniformity in non-uterine large-organ models, their application to intact uterine cryopreservation remains investigational. Collectively, current evidence supports uterine cryopreservation, particularly in porcine models, as a preclinical, hypothesis-generating framework for whole-organ preservation research rather than a clinically established strategy. Given the absence of successful clinical transplantation of any cryopreserved solid organ and the lack of reproductive outcome data, findings should be interpreted cautiously. Continued standardized large-animal studies incorporating functional, vascular, and reproductive endpoints are essential to further define this frontier field before uterine organ banking or any clinical application can be responsibly considered.

## Introduction and background

Solid-organ transplantation, first achieved clinically in 1954 with the successful renal transplant performed in Boston [1], has since become a cornerstone therapeutic strategy for restoring organ function and improving survival and quality of life worldwide [2]. However, the prevailing donation model based on brain-dead donors (BDDs) remains constrained by immunological incompatibility, logistical barriers related to geographic distance, and limited tolerance to cold ischemia, leading to the loss of potentially viable grafts even in advanced healthcare systems [3].

To overcome these limitations, cryopreservation has emerged as a promising experimental strategy to extend extracorporeal organ viability by inducing a state of suspended biological activity. Unlike approaches such as ex vivo perfusion or xenotransplantation, cryopreservation enables the concept of organ banking, facilitates pre-transplant immunological assessment, and reduces spatial and temporal barriers to allocation [4]. Recent advances in rewarming technologies, including nanowarming, have further strengthened this concept by mitigating rewarming injury and improving thermal uniformity in large tissue volumes under experimental conditions [5].

Cryobiological approaches have demonstrated structural preservation and partial functional recovery across a range of biological scales and animal models, including small-organ systems and large-tissue applications in porcine cartilage and kidney models [6]. Nevertheless, translation of whole-organ cryopreservation to routine clinical practice remains limited by ethical constraints and restricted access to human organs for experimental research. Accordingly, no solid organ has yet been successfully cryopreserved, transplanted, and shown to sustain long-term functional outcomes in humans, underscoring the experimental nature of this field [7].

Within this context, the uterus has emerged as an ethically acceptable and experimentally tractable model for the development of whole-organ cryopreservation protocols. Its anatomical and functional complexity, comparable in several respects to vital organs, combined with its non-vital status, makes it particularly suitable for methodological research focused on organ preservation and banking [8]. Among available experimental systems, the porcine uterus is especially valuable due to its close similarity to the human organ in terms of size, histological architecture, vascular organization, and hormonal responsiveness, and has therefore played a central role in protocol optimization [9]. Studies employing perfusion-based slow cryopreservation with dimethyl sulfoxide (DMSO) and controlled cooling have reported preservation of histological integrity and partial post-thaw contractile activity in porcine uterine tissue, supporting its role as a robust preclinical experimental platform rather than evidence of direct clinical applicability [10].

At the same time, it is important to recognize that evidence supporting the cryopreservation of intact organs remains scarce, and that whole-organ preservation is constrained by fundamental technical limitations related to organ size, structural heterogeneity, and complex vascular architecture [6,7]. These constraints complicate uniform cryoprotectant delivery, heat transfer, and controlled rewarming, even in advanced experimental systems. In the uterus, such challenges are further amplified by heterogeneous perfusion between myometrial and endometrial compartments, steep internal thermal gradients, and cryoprotectant-related toxicity, as observed in large-animal experimental models [9,10]. Accordingly, uterine cryopreservation should be regarded as a frontier area of investigation, in which current findings provide preclinical, hypothesis-generating insight rather than evidence of immediate clinical applicability [7,11].

Against this background, this review synthesizes current freezing and thawing strategies applied to the porcine uterus to integrate available preclinical evidence, identify reproducible experimental patterns, and define unresolved challenges in uterine cryopreservation. In the absence of reported clinical cases demonstrating functional reproductive outcomes after uterine cryopreservation and transplantation, continued experimental research remains essential to further characterize this frontier field and to establish the scientific foundations required before human organ banking can be responsibly considered [11].

## Review

Methodology

This review examines the evolution, current progress, and emerging perspectives of uterine cryopreservation in animal models, with particular emphasis on experimental in vitro and ex vivo studies. The objective was to synthesize available preclinical evidence on strategies aimed at preserving the structural and functional integrity of uterine tissue, and to contextualize these approaches within the broader field of organ cryobiology and uterine transplantation research.

A structured literature search was conducted across PubMed/MEDLINE, Embase, Web of Science, Scopus, the Cochrane Library, and LILACS, covering records published up to October 2025, without restrictions on language or publication date. Searches combined controlled vocabulary and free-text terms related to uterus, cryopreservation, vitrification, slow freezing, cryoprotectants, and animal models. Reference lists of relevant articles were also manually screened to identify additional studies of potential relevance.

Eligible studies consisted of experimental research using animal models in which cryopreservation protocols were applied to whole uteri or representative uterine segments under controlled laboratory conditions. Studies were considered relevant if they included post-thaw assessment of tissue viability based on histological, cellular, or functional parameters. Research focused exclusively on gametes, embryos, or non-uterine tissues, as well as narrative reviews, clinical reports, or studies lacking post-thaw evaluation, were excluded from the synthesis.

Study selection and data extraction were performed by two reviewers with expertise in cryobiology and experimental surgery, with discrepancies resolved by consensus. Extracted information included animal species, general experimental design, cryoprotectant composition, cooling and warming approaches, and reported histological or functional outcomes. Given the heterogeneity of experimental designs, preservation protocols, and outcome measures, findings were integrated using a qualitative descriptive synthesis, rather than a quantitative or meta-analytic approach.

An initial pool of 43 publications was identified through the search process. Following relevance screening and qualitative appraisal of methodological reporting, 29 studies were retained for inclusion. Studies were excluded primarily because of insufficient methodological description, absence of post-thaw outcome assessment, or limited relevance to uterine tissue preservation.

Methodological considerations related to animal experimentation were interpreted with reference to the ARRIVE guidelines as a framework for transparency and reporting quality, without formal scoring or comparative risk of bias assessment. Ethical approval was not required, as this review was based exclusively on previously published data. Collectively, the selected studies illustrate the progressive experimental development of uterine cryopreservation, from early slow-freezing approaches in small-animal models to more advanced perfusion-based strategies in large animals, providing a preclinical and hypothesis-generating foundation for future research.

Historical development of cryopreservation

Cryopreservation is a biomedical technique that enables the preservation of cells, tissues, and organs by cooling them to extremely low temperatures, typically below −130°C. The primary objective of this strategy is to halt metabolism and biological activity, thereby allowing long-term storage of biological material without inducing deleterious structural or biochemical alterations. This technology has gained growing importance in key areas such as regenerative medicine, reproductive biology, biobanking, and organ transplantation [12-14].

In parallel, several organisms in nature have evolved adaptive mechanisms that allow them to withstand freezing conditions. These species serve as valuable biological models for the design of artificial cryopreservation strategies. For instance, the wood frog (*Rana sylvatica*) survives the freezing of a substantial fraction of its body water by accumulating endogenous cryoprotectants such as glucose and urea, which stabilize cellular membranes during winter dormancy [15]. Similarly, the Arctic beetle larva *Upis ceramboides* synthesizes trehalose and antifreeze proteins that prevent ice crystal formation and preserve cellular integrity even at subzero temperatures [16]. Other naturally cryotolerant organisms include Antarctic fish, reptiles such as *Thamnophis sirtalis*, nematodes, and tardigrades, species capable of surviving extended periods of anhydrobiosis through metabolic suppression and structural stabilization mechanisms [17].

These biological adaptations have inspired the development of modern cryopreservation technologies. Throughout the twentieth century, progressive advances in controlled-rate cooling and the introduction of cryoprotective agents, most notably DMSO, glycerol, trehalose, and various sugar-based compounds, enabled successful preservation of isolated cells and tissues in vitro [18-20]. Refinements in these techniques led to the reliable cryostorage of embryos, hematopoietic stem cells, and reproductive tissues, establishing the scientific foundation of contemporary cryobiology and biobanking [21-23].

Despite these achievements, the cryopreservation of whole organs remains a formidable technical challenge due to their large size, heterogeneous structure, and vulnerability to both thermal and osmotic stresses. Nonetheless, important progress has been achieved in animal models. In rabbits, the successful cryopreservation and autologous transplantation of ovaries have demonstrated recovery of endocrine and reproductive function after thawing [24].

Similarly, experimental work in rodent models has achieved partial reimplantation and functional recovery of vitrified kidneys following nanoparticle-assisted rewarming, illustrating the translational potential of these emerging techniques [25].

In pigs, an anatomically relevant species due to its close similarity to humans, research has primarily focused on the preservation of cardiac, hepatic, and renal tissues [26]. Recent experimental evidence demonstrates that rapid and uniform rewarming techniques, such as radiofrequency heating combined with magnetic nanoparticles, can substantially reduce recrystallization-associated injury during the thawing process, improving post-thaw structural preservation and functional viability [26].

These preclinical advances not only bring the field closer to achieving long-term human organ storage but also position cryopreservation as a fundamental tool for extending the viable time window between organ retrieval and transplantation. The convergence of natural cryotolerance mechanisms with modern developments in biomedical engineering, including nanowarming and optimized cryoprotectant systems, represents a promising strategy to overcome the current physiological and logistical barriers in transplant medicine [26].

Application of the laws of thermodynamics in cryopreservation

Cryopreservation is a core biomedical technology that enables the preservation of biological tissues and whole organs through controlled cooling to ultra-low temperatures, typically below −130°C [11]. Its main objective is to suspend metabolic activity and biochemical reactions without inducing irreversible cellular or molecular damage, a prerequisite for maintaining the structural and functional viability of complex tissues [5,11].

This process is grounded in the principles of thermodynamics, which describe the behavior of matter and energy during temperature and phase transitions. These principles are crucial for understanding and optimizing each stage of the cryopreservation process, from cooling to rewarming [11,12].

According to the zeroth law of thermodynamics, if system A is in thermal equilibrium with system B, and system B is in equilibrium with system C, then A and C are also in equilibrium. This principle is fundamental in cryopreservation, ensuring that the tissue, cryoprotectant solution, and external medium reach thermal equilibrium before cooling below freezing temperatures. For instance, during rabbit ovary cryopreservation, establishing such an equilibrium between the tissue and the cryoprotectant is essential to prevent uneven ice nucleation that could compromise follicular integrity at the eutectic point [12,21].

The first law of thermodynamics (law of energy conservation) states that the internal energy of a system changes according to heat exchange and work performed. During cooling, the extraction of thermal energy lowers molecular kinetic activity, reducing temperature and slowing metabolism. In mammalian embryos, this process inhibits biochemical reactions while maintaining cellular viability, effectively creating a closed system that exchanges energy but not matter with its surroundings [11,15].

The second law of thermodynamics establishes that entropy, or molecular disorder, tends to increase spontaneously. Cryopreservation counteracts this tendency by reducing temperature, thereby limiting molecular motion and stabilizing cellular structures. If cooling is too slow, however, ice nucleation occurs, i.e., the ordered crystallization of water molecules, which produces mechanical and osmotic damage. To mitigate this effect, cryoprotectants such as DMSO, trehalose, and deep eutectic solvents are employed to induce vitrification, an amorphous glass-like state that avoids crystal formation and preserves tissue microarchitecture [4,5,13].

In porcine ovarian tissues, for example, these vitrification systems have demonstrated effective prevention of ice crystal formation and preservation of histological integrity during freezing and thawing cycles [13,25,26].

The third law of thermodynamics states that as a system approaches absolute zero (0 K), the entropy of a perfectly ordered system approaches zero. Although cryopreservation temperatures do not reach this absolute limit, they effectively minimize entropy by immobilizing molecular motion and inducing an amorphous, vitrified structure that halts all biochemical reactions [11,15].

This molecular immobilization allows for long-term organ storage, as demonstrated in vitrified porcine livers, where preservation of cellular architecture and partial restoration of metabolic function have been observed upon controlled and uniform rewarming using superparamagnetic nanoparticles or radiofrequency heating techniques [3,8,9].

Throughout the cryopreservation process, the biological system can behave as open, closed, or nearly isolated, depending on its stage. During the introduction of cryoprotectants, active mass transfer occurs, rendering the system open. During cooling and maintenance at subzero temperatures, matter exchange ceases, but energy transfer continues, characterizing a closed system. Finally, during long-term storage in liquid nitrogen, both matter and energy exchanges are minimized, approximating an almost isolated system that ensures thermodynamic stability of the stored organ [11,15].

In summary, the application of thermodynamic principles provides a robust theoretical framework to understand, optimize, and predict biological behavior during cryopreservation. When integrated with physicochemical approaches such as vitrification and emerging rewarming technologies such as nanowarming, these principles contribute to more effective preservation of animal tissues and support translational progress toward clinical organ cryopreservation and transplantation [5,9,10,15].

Modulation of nucleation and glass transition by cryoprotectants and eutectic solutions during cryopreservation

From a physicochemical perspective, biological systems can be conceptualized as complex aqueous matrices composed of electrolytes, proteins, carbohydrates, lipids, and other solutes dissolved in water. Depending on their viscosity and molecular organization, these systems can be classified as cytosols, characterized by low viscosity and high molecular mobility, or cytogels, which display denser molecular networks and restricted mobility [16,24,25]. This distinction is crucial in cryopreservation, as water mobility and solute dynamics directly influence nucleation behavior and the capacity to achieve a stable glassy state during cooling [16,25].

Nucleation, defined as the initial aggregation of water molecules into an ordered ice crystal lattice, represents a critical thermodynamic event since intracellular nucleation can result in irreversible cellular and structural injury. Cryoprotective agents modulate this process by increasing viscosity, lowering the freezing point, and altering hydrogen bonding, thereby delaying or preventing ice crystal formation [4,5,13].

Furthermore, the use of deep eutectic solvents as alternative cryoprotectants has shown promise in regulating both nucleation and the glass transition temperature, offering enhanced stability and reduced toxicity compared with conventional cryoprotective agent systems [5,21,24].

The viscosity of the cryoprotective medium is a critical parameter in cryopreservation. At low temperatures, increased viscosity limits molecular mobility and promotes vitrification, a process associated with the glass transition, in which the solution transforms into a solid amorphous state without ice crystallization [4,15,17]. Upon reaching the glass transition temperature (Tg), the aqueous matrix becomes immobilized in a disordered configuration that prevents molecular rearrangement of water molecules. However, excessive viscosity can hinder the homogeneous diffusion of cryoprotectants within tissues, thereby compromising the overall efficacy and uniformity of the freezing process [17,26].

A historic milestone in cryobiology was achieved by Basile Luyet in the 1930s, who demonstrated that supersaturated sugar solutions could undergo vitrification through rapid cooling, thereby avoiding ice crystal formation. This seminal observation introduced the concept of the glass transition and established the theoretical basis for contemporary vitrification techniques employed in reproductive and organ preservation research [17,25].

In this context, eutectic solutions have become particularly relevant. These are homogeneous mixtures of water and solutes, such as salts, sugars, or cryoprotectants, that solidify as a single phase upon reaching their eutectic point. Unlike fractional freezing, eutectic systems prevent selective crystallization of individual components, promoting more uniform solidification and reducing structural damage during cooling [5,18,21]. For instance, a glycerol-water eutectic mixture exhibits a freezing point significantly lower than that of pure water, thereby minimizing the risk of premature nucleation and facilitating more stable vitrification dynamics [18,23,25].

During freezing, the plasma membrane, a semipermeable barrier, plays a fundamental role in maintaining intracellular osmotic equilibrium. Under physiological conditions, this balance is sustained by ATP-dependent transport mechanisms. However, thermal descent during cryopreservation inhibits enzymatic activity, disrupts ion homeostasis, and increases the risk of intracellular nucleation and structural injury [4,19]. To counteract these effects, cryoprotective agents are employed to modulate the freezing point, control osmotic gradients, and preserve cellular integrity by preventing ice crystal formation and membrane rupture [4,5,13,15,19].

Cryoprotectants are typically classified as permeating or non-permeating agents (Table 1). Their combined or sequential use depends on the cell type, cooling rate, cryoprotectant concentration, and intended storage duration, allowing customized optimization for each biological system and experimental protocol [4,5,19,26].

**Table 1:** Comparison of permeable and non-permeable cryoprotective agents: molecular weight, viscosity, and osmolarity. Osmolarity varies depending on concentration and combination with other cryoprotectants. The indicated values are approximate and commonly used in cryopreservation protocols. • Viscosity values correspond to pure or concentrated solutions at 20°C and are estimates; in multicomponent mixtures, they may change significantly. • The physiological osmolarity for human cells is approximately 280–300 mOsm/kg; cryoprotective solutions are intentionally hyperosmotic to induce controlled cellular dehydration. DMSO = dimethyl sulfoxide; CPA = cryoprotectant agent; HES = hydroxyethyl starch; PEG 400 = polyethylene glycol 400 g/mol; PEG = polyethylene glycol 8,000 g/mol

Type	Example	Molecular weight (g/mol)	Viscosity at 20°C (mPa·s)	Approximate osmolarity (typical concentration)	Main characteristics	Study/Author
Permeable	DMSO	78.1	~2.0	~1,400 mOsm/kg (10% v/v)	High permeability, depresses freezing point, intracellular action	Taylor et al. (2019) [1]
	Glycerol	92.1	~1.5	~1,200 mOsm/kg (10% v/v)	Stabilizes membranes; widely used in cells, embryos, tissues	Taylor et al. (2019) [1]
	Ethylene glycol	62.1	~16.1	~1,500 mOsm/kg (10% v/v)	High permeability; effective for vitrification of oocytes/embryos	Murray and Gibson (2022) [2]
	1,2-Propanediol	76.1	~40.0	~1,250 mOsm/kg (10% v/v)	Good cellular tolerance; commonly used for vitrification	Taylor et al. (2019) [1]
Non-permeable	Sucrose	342.3	50–60 (50% w/w)	~850 mOsm/kg (0.2 M)	Osmotic dehydrator; extracellular CPA; membrane stabilizer	Fahy et al. (1984) [25]
	Trehalose	342.3	Similar to sucrose	~850 mOsm/kg (0.2 M)	Protects proteins; promotes vitreous state; intracellular uptake via carriers	Fujita et al. (2022) [14]
	PEG 400	~400	90–100	200–400 mOsm/kg (5–10% w/v)	Non-permeating polymer; increases viscosity; additive for vitrification	Chen et al. (2023) [13]
	PEG 8000	~8000	400–600 (30% w/v)	100–200 mOsm/kg (5% w/v)	Extracellular action; reduces free water; enhances vitrification	Chen et al. (2023) [13]
	HES	130,000–200,000	>1,000 (6% w/v)	150–300 mOsm/kg (6% w/v)	Highly viscous macromolecule; stabilizes membranes/structures; vitrifier	Fahy et al. (1984) [25]

In closing, the efficacy of cryopreservation depends on the precise and balanced formulation of cryoprotective solutions, which must account for factors such as molecular weight, viscosity, solubility, toxicity, and their interactions with cellular membranes and intracellular macromolecules [4,5,15].

Furthermore, understanding and controlling fundamental physicochemical processes, including nucleation dynamics, the eutectic point, and the glass transition phenomenon, is essential to achieving optimal outcomes in advanced biomedical applications such as regenerative medicine, reproductive biology, and organ transplantation [16,17,18].

Influence of cooling rate and duration during cryopreservation processes

In cryopreservation, accurate control of cooling rate and exposure duration is critical for maintaining cellular viability, as these parameters directly influence the processes of nucleation, eutectic crystallization, and the glass transition [15-17].

The cooling rate, defined as the speed of temperature reduction (°C/minute), determines the time that biological material remains within thermal intervals favorable for ice crystal formation or eutectic solidification. High cooling rates minimize exposure to these unstable zones, while slow cooling rates prolong it, promoting undesirable events such as nucleation, solute precipitation, and intracellular dehydration [17,21,24].

From a thermodynamic perspective, the first law of thermodynamics, i.e., the principle of energy conservation, explains that cooling a system decreases its internal energy. However, this process simultaneously releases latent heat of crystallization, which can locally slow further cooling if not efficiently dissipated, particularly in multicellular tissues where heterogeneous heat transfer limits uniform solidification [11,15,22].

Similarly, the second law of thermodynamics states that entropy, or molecular disorder, tends to increase spontaneously. Nevertheless, crystallization, representing a more ordered state, may occur when temperature decreases slowly enough to permit molecular alignment. Thus, slow cooling promotes structural ordering of water molecules and facilitates ice nucleation, whereas rapid cooling suppresses this reorganization by reducing molecular mobility, favoring the formation of an amorphous vitrified state [15,17,21].

For example, in human oocytes, cooling rates exceeding 10,000°C/minute have been shown to prevent intracellular nucleation and markedly improve post-thaw viability and structural preservation [14,21].

Moreover, intracellular ice formation, which arises from prolonged exposure to nucleation-prone temperature ranges, is one of the principal causes of cellular damage during freezing. Reducing this exposure window through rapid cooling prevents sufficient molecular order for ice nucleation, forcing the system into a non-crystalline amorphous phase that protects subcellular structures from mechanical and osmotic stress [21,22,24].

This principle also extends to the prevention of eutectic crystallization. During slow cooling, water tends to solidify before solutes, increasing solute concentration and promoting the formation of eutectic solids that generate osmotic injury. Accelerated cooling, by contrast, avoids the equilibrium conditions necessary for eutectic crystal development. This effect has been demonstrated in murine embryos, where preventing salt crystallization during rapid cooling significantly improved post-thaw recovery and developmental potential [22,24,25].

Finally, the effective freezing duration, defined as the interval during which the sample passes through critical thermal transition zones, must be carefully optimized. Excessively rapid freezing can induce thermal and osmotic shock if temperature gradients are uncontrolled, while overly slow freezing extends exposure to conditions favoring crystal growth and solute-induced dehydration.

**Figure 1:**
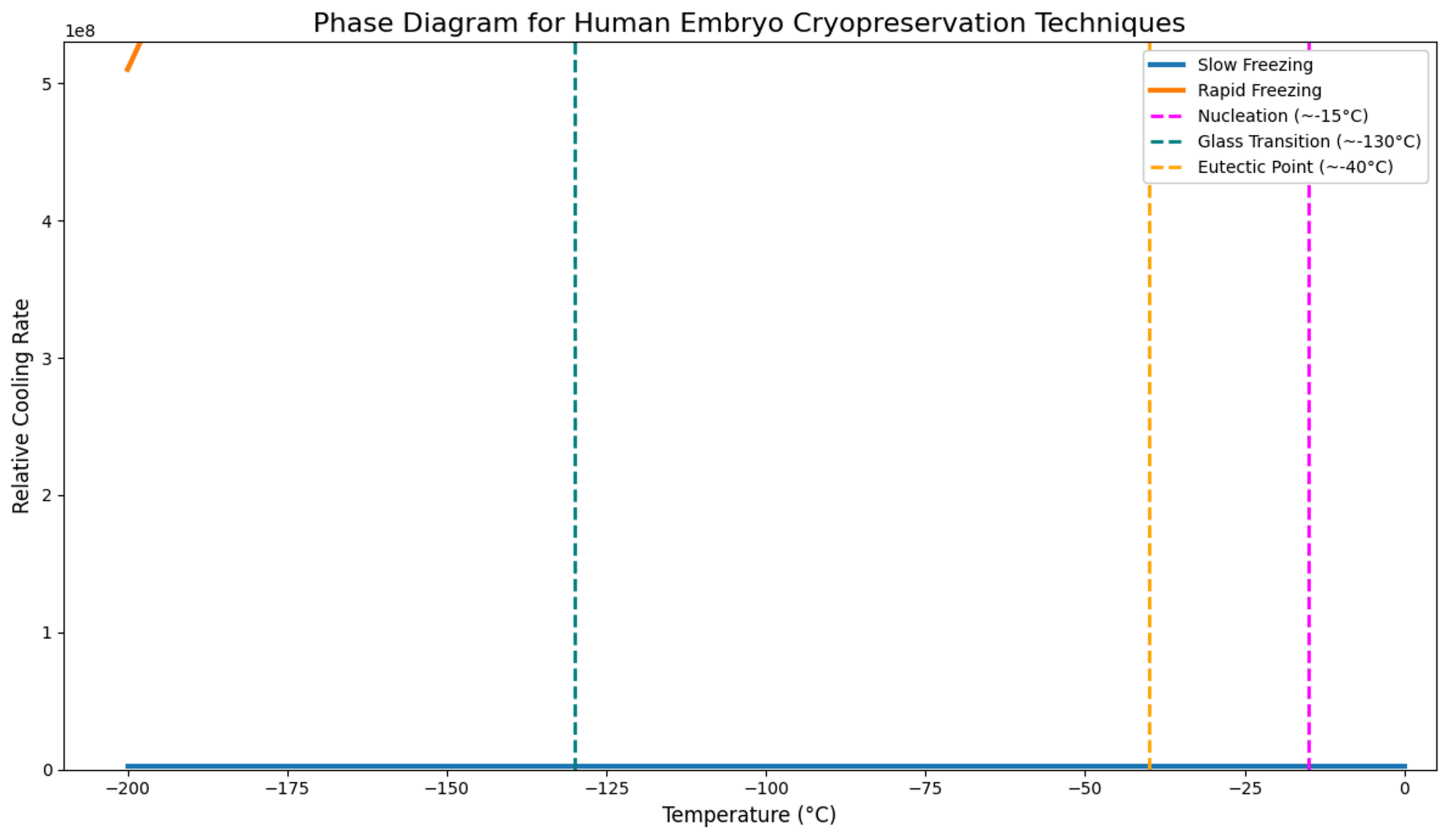
Phase diagram of embryonic cryopreservation: comparison between slow and rapid techniques. The diagram compares slow freezing and vitrification in human embryos. Slow freezing promotes ice crystal formation around –15°C, which can damage cells. Vitrification prevents crystal formation by rapidly reaching a glassy state below –130°C, thereby improving viability. The eutectic point (~–40°C) indicates the crystallization of water and salts, whose control is crucial to prevent osmotic damage.

In clinical ovarian vitrification, for instance, the use of supersaturated cryoprotectant mixtures in combination with optimized cooling and warming rates has proven effective in minimizing these risks. This strategy enables rapid traversal through the eutectic temperature zone, preventing nucleation and ensuring preservation of tissue architecture and cellular functionality after thawing [14,23,24] (Figure 1).

Cryopreservation techniques

Cryopreservation, understood as the process of preserving cells, tissues, or organs at extremely low temperatures, has established itself as a fundamental tool in biomedicine, particularly in the fields of assisted fertility, regenerative medicine, and genetic resource conservation [4-6]. Its primary goal is to prevent intracellular ice formation, which constitutes the main cause of cellular damage during cooling [9,14,15]. To achieve this, two main methodologies have been developed: slow freezing and vitrification. Both present advantages and limitations that merit detailed comparison to determine the most suitable option depending on the clinical or experimental context [17,24,25].

First, it is necessary to consider the complexity of the process and the associated costs of each technique. Slow freezing involves a gradual decrease in temperature at an approximate rate of 1°C per minute, using relatively low concentrations of cryoprotective agents, generally below 1 M [15,17,21]. This procedure requires specialized equipment such as programmable freezers or freezing trays, which increases the initial cost. However, its execution is usually simpler and less dependent on operator skill, representing an advantage in settings with limited technical expertise [21,24].

In contrast, vitrification relies on much higher cryoprotective agent concentrations (between 40% and 60%) and extremely rapid cooling rates, exceeding 10,000°C per minute [14,17,25]. Although this technique allows the process to be completed within minutes and does not require complex equipment, it demands a high degree of operator skill due to the speed and precision necessary to avoid ice formation. Additionally, the intensive use of cryoprotectants can induce cellular toxicity if not properly managed, representing an additional challenge [4,5,25]. Nevertheless, operational costs may be lower due to the absence of programmable equipment [25].

From a physicochemical perspective, the two techniques differ significantly in terms of nucleation, eutectic point, and glass transition [16,17,18]. In slow freezing, the gradual temperature decrease leads to extracellular ice formation, a process initiated at the eutectic point [9,17,22]. Although controlled, this phenomenon can trigger nucleation and cellular damage due to osmotic dehydration and mechanical stress generated by the crystals [14,15,17]. Vitrification, on the other hand, aims to completely avoid nucleation by maintaining water in an amorphous or glassy state [4,5,13]. This glass transition prevents crystallization and, consequently, eliminates mechanical damage associated with ice, enabling more efficient preservation of cellular and subcellular structures (Table 2) [8,9,13].

**Table 2:** Comprehensive comparison of slow freezing and vitrification: physicochemical, operational, and clinical considerations.

Process/Characteristic	Time-dependent	Slow freezing	Vitrification (rapid freezing)	Study/Author
Nucleation	Yes	High nucleation → intra/extracellular crystals → mechanical damage	Low/absent nucleation → amorphous glass formation → prevents crystal damage	Fahy et al. (1984) [25]
Eutectic point	Yes	May be reached → salt crystallization risk	Avoided with ultrarapid cooling → no crystallization	Fahy et al. (1984) [25]
Glass transition	Yes	Not reached → water crystallizes as ice	Reached → vitreous state	Taylor et al. (2019) [1]
Process speed	Yes	Slow (hours)	Fast (minutes)	Chen et al. (2023) [13]
Cryoprotectant (CPA) concentration	Yes	Low (<1 M) → low toxicity	High (40–60%) → higher toxicity	Murray and Gibson (2022) [2]
Technical complexity	Yes	Low complexity	High operator-dependence	Chen et al. (2023) [13]
Technological requirements	Partially	Programmable freezers required	Not equipment-dependent	Taylor et al. (2019) [1]
Costs	Partially	High equipment costs, low CPA use	Low equipment cost, high CPA use	Chen et al. (2023) [13]
Cellular toxicity	No	Low toxicity	High toxicity (CPA concentration)	Murray and Gibson (2022) [2]
Post-thaw cell viability	Yes	Lower viability (ice-induced damage)	Higher viability (membrane protection)	Taylor et al. (2019) [1]
Common clinical applications	Yes	Complex tissues (ovarian cortex, cord tissue)	Oocytes, embryos, stem cells	Behl et al. (2023) [20]
Results in assisted fertility	Yes	Lower survival (oocytes ~61%, embryos ~91%)	Higher survival (oocytes ~91%, embryos ~98%)	Behl et al. (2023) [20]
Examples of efficacy	Yes	Lower stromal viability (48%)	Higher stromal viability (60%)	Karimi et al. (2023) [11]
Current trend	Yes	Declining for single cells but used for complex tissues	Preferred globally for assisted fertility	Chen et al. (2023) [13]
Future perspectives	Yes	Protocol optimization, nucleation control, hybrid techniques	—	Taylor et al. (2019) [1]

To sum up, while traditional techniques such as convection and conduction offer accessible but limited solutions, nanowarming represents a significant advance for ensuring cellular integrity during thawing [2,13,14]. Controlling not only temperature but also time and uniformity in this process is essential to maximize the benefits of cryopreservation and achieve successful outcomes in biomedical applications [5,7,9].

Porcine uterus cryopreservation

Experimental evidence on uterine cryopreservation at the organ level remains limited and is derived predominantly from studies using the porcine model, which poses specific challenges due to the organ’s size, vascular complexity, and functional demands [11,13,14]. The anatomical and physiological similarity between the porcine and human uterus has nevertheless established this model as a relevant preclinical platform for evaluating preservation strategies applicable to translational research [23-25].

Across the reported studies, uterine preservation protocols commonly incorporated an initial phase of hypothermic perfusion before cryopreservation. This intermediate step, performed at approximately 4°C, aimed to reduce metabolic activity and attenuate ischemia-related injury [7,11,23]. In porcine uteri, continuous low-temperature perfusion using solutions such as Custodiol® HTK or Perfadex®, often supplemented with antioxidants and oncotic agents, preserved endothelial integrity for periods of up to 24 hours [23,25]. Perfusion was typically conducted via the uterine arteries under controlled pressures (30-50 mmHg), facilitating homogeneous vascular distribution without structural compromise [23,24].

Following perfusion, cryopreservation was achieved exclusively through slow-freezing protocols in the available porcine studies. These protocols involved progressive cooling from hypothermic conditions, exposure to permeable cryoprotectants such as DMSO and ethylene glycol, and the addition of non-permeable agents, including sucrose or albumin [14,15,17,21]. Temperature reduction was generally performed at controlled rates (−0.3 to −1°C/minute) before storage at cryogenic temperatures [14,17]. Post-thaw evaluation demonstrated preservation of uterine histological architecture and retention of ex vivo myometrial contractile activity, although contraction amplitude was consistently reduced and biochemical markers indicated residual cellular stress [27-29]. A structured summary of these experimental protocols, cryopreservation techniques, and functional outcomes is provided in Table 3.

**Table 3:** Experimental studies on uterine cryopreservation and functional outcomes Only one study evaluated cryopreservation of a whole porcine uterus. Earlier studies were limited to uterine segments from small-animal models. No study included vitrification, transplantation, or reproductive outcomes CPA = cryoprotective agent; DMSO = dimethyl sulfoxide; PGF2α = prostaglandin F2 alpha

Model	Tissue scope/Sample size	Preservation technique	Cryoprotectant (CPA)	Cooling/Rewarming	Viability outcomes	Contractility assessment	Author (year)
Rat uterus	Uterine segments; n≈20	Slow freezing	Permeable CPA (DMSO-based)	Controlled-rate cooling; water-bath thawing	Preserved histological architecture	In vitro spontaneous and oxytocin-induced contractions preserved	Dittrich et al. (2006) [27]
Rat uterus	Uterine segments; n≈80	Slow freezing	DMSO or glycerol	Controlled cooling; conventional thawing	Acceptable tissue survival with biochemical alterations	Spontaneous and pharmacologically induced contractility maintained	Dittrich et al. (2010) [28]
Porcine uterus	Whole uterus; 60 cryopreserved, 15 fresh controls	Slow freezing (perfusion-based)	DMSO-based CPA	Slow freezing; short- and long-term storage; bath thawing	Preserved histology with mild subclinical stress	Reproducible contractions to oxytocin and PGF2α, reduced amplitude	Schölch et al. (2012) [29]

**Figure 2:**
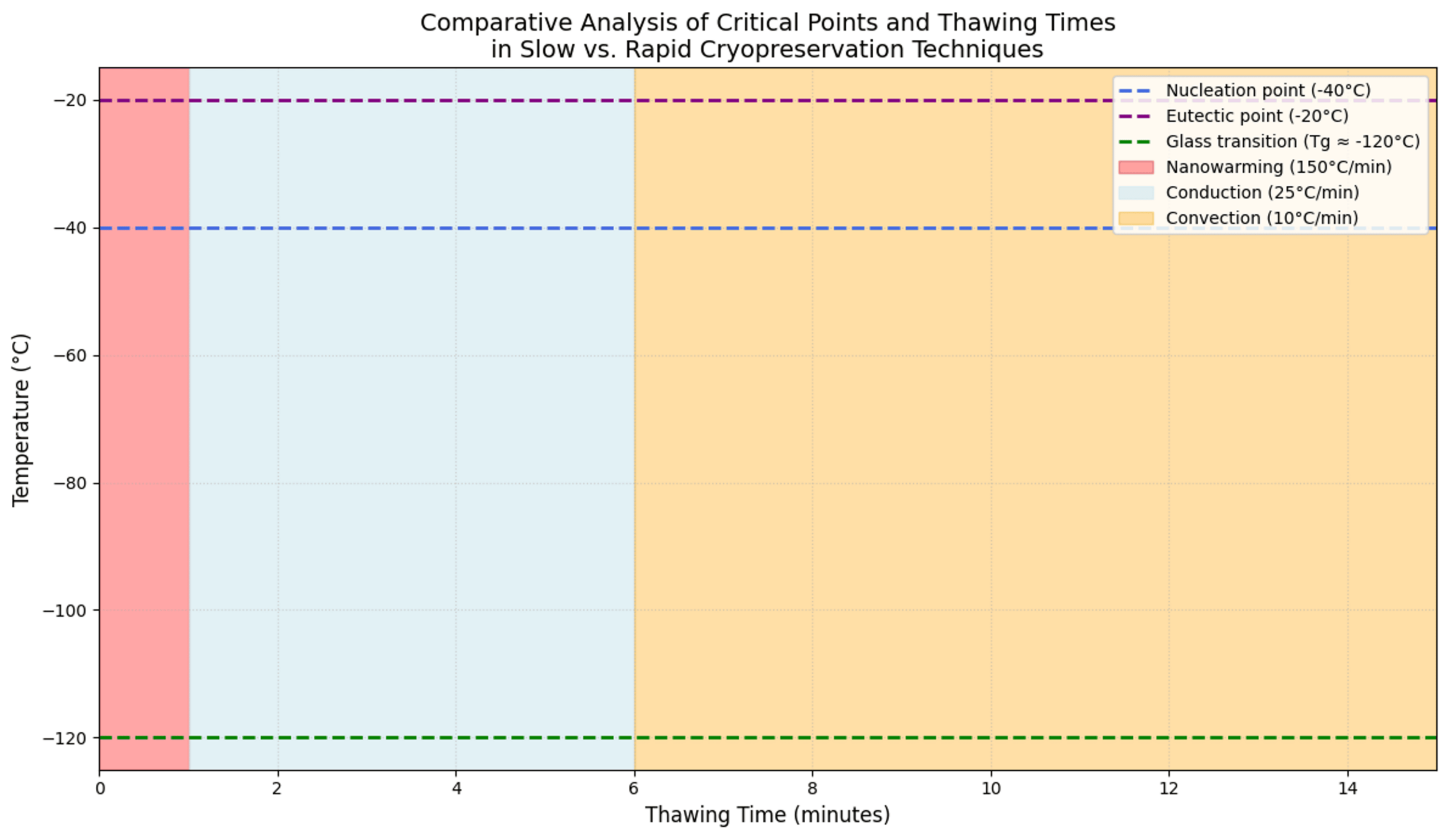
Comparative evaluation of thawing techniques and their critical points in the context of cryopreservation. The colored bands in the diagram represent the thawing techniques, i.e., convection (orange), conduction (light blue), and nanowarming (red), with progressively increasing heating rates from –150°C to 0°C. These rates determine the duration that the sample remains within critical temperature zones, such as nucleation (–40°C), eutectic point (–20°C), and glass transition (–120°C), influencing ice crystal formation and, consequently, cell viability. Thus, faster techniques such as nanowarming minimize thermal damage by reducing the exposure time within these critical temperature regions.

Rewarming procedures varied among studies but typically involved rapid immersion in isothermal baths followed by sequential washout of cryoprotectants to minimize osmotic injury [14,23]. Conventional rewarming approaches were limited by relatively slow heating rates, which may prolong tissue exposure to critical temperature ranges associated with recrystallization [2,3,7]. Advanced internal heating strategies, such as nanowarming, have been proposed to address these limitations by enabling more homogeneous heat distribution in large tissue volumes (Figure 2). However, although nanowarming has shown promising results in non-uterine large-organ models, its application to intact porcine uteri has not yet been systematically evaluated, and no comparative data between slow freezing and vitrification at the whole-organ uterine level are currently available [2-5,8].

Some studies incorporated post-thaw ex vivo normothermic perfusion to assess functional viability, maintaining uterine tissue at physiological temperature with oxygenated perfusates [7,9,23]. Under these conditions, preserved uterine tissue demonstrated measurable contractile responses and metabolic activity for several hours, allowing verification of organ viability following cryopreservation [11,23,26]. However, these assessments remained limited to short-term functional endpoints and did not include transplantation or reproductive outcomes.

Overall, the reported results indicate that porcine uterine cryopreservation using perfusion-assisted slow freezing can preserve structural integrity and partial contractile function ex vivo. At the same time, the absence of vitrification-based organ-level studies, the lack of direct technique comparisons, and the restriction of outcomes to short-term functional measures underscore the experimental and exploratory nature of current evidence.

Discussion

Cryopreservation of solid organs remains one of the most complex and unresolved challenges in contemporary translational and preclinical research [1,6,9]. Its development is driven by the need to overcome fundamental limitations of current transplantation paradigms, including organ scarcity, restricted ischemia tolerance, and logistical and immunological constraints that persist despite advances in surgical technique and perioperative management [7,9,10]. Unlike isolated cells or thin tissues, whole-organ preservation requires the simultaneous maintenance of structural integrity, vascular patency, and functional responsiveness throughout the entire freezing and rewarming process, substantially increasing technical complexity and limiting immediate clinical applicability [8,9,11].

At present, experimental cryopreservation of human organs remains ethically and legally constrained. The use of viable organs for research conflicts with principles of distributive justice, which prioritize allocation to patients awaiting transplantation [18]. In parallel, regulatory frameworks in many jurisdictions prohibit the diversion of donated organs for non-therapeutic experimental purposes, except under highly restricted conditions [18,19]. These limitations underscore the central role of animal models in advancing organ cryopreservation research.

Within this context, large-animal models, and particularly the porcine uterus, have emerged as essential experimental platforms [9,10]. The porcine uterus shares key anatomical, histological, and functional features with the human organ, including comparable size, endometrial organization, vascular architecture, and hormonal responsiveness [11,13,14,23-25]. These characteristics make the porcine model especially suitable for evaluating perfusion strategies, cryoprotectant delivery, and post-thaw functional assessment under conditions that more closely approximate human physiology than small-animal systems [23,24,26].

The results synthesized in this review indicate that slow freezing remains the only cryopreservation strategy that has been systematically evaluated at the level of the intact porcine uterus. Controlled-rate cooling combined with permeable cryoprotectants such as DMSO and ethylene glycol has been shown to preserve overall tissue architecture and endothelial markers, and to maintain partial myometrial contractility following thawing [14,15,17,23,29]. However, even within these protocols, reduced contraction amplitude and biochemical indicators of cellular stress have been consistently observed, highlighting incomplete functional preservation [29].

In contrast, vitrification, although conceptually attractive due to its ability to prevent ice crystal formation, has not yet been robustly applied or validated in intact uterine organs. Existing evidence supporting vitrification and advanced rewarming strategies such as nanowarming derives primarily from non-uterine large-organ models or from smaller tissue systems [2-5,8]. Consequently, extrapolation of these approaches to the uterus must be undertaken cautiously, particularly given the organ’s heterogeneous perfusion, complex three-dimensional structure, and sensitivity to cryoprotectant toxicity [13,14,17].

Post-thaw evaluation strategies further illustrate the preclinical nature of current evidence. Functional assessment has largely been limited to ex vivo measures, such as myometrial contractility in response to uterotonic agents and short-term metabolic activity during normothermic perfusion [7,9,11,23,26]. While these endpoints provide important proof-of-concept data, they do not address longer-term outcomes critical for translation, including vascular integrity after reperfusion, endometrial receptivity, hormonal responsiveness, placentation, or reproductive capacity following transplantation.

From an ethical and translational standpoint, the uterus occupies a unique position among solid organs. As a non-vital organ with profound functional relevance in reproductive medicine, it offers an ethically acceptable platform for developing and validating cryopreservation technologies that may ultimately inform preservation strategies for vital organs [18,23,25]. However, this role should be understood as experimental rather than indicative of imminent clinical application.

Taken together, the findings reviewed here support uterine cryopreservation, particularly in porcine models, as a preclinical, hypothesis-generating framework for advancing whole-organ preservation science. At the same time, the absence of direct comparisons between slow freezing and vitrification in intact uteri, the lack of transplantation and reproductive outcome data, and the persistence of unresolved technical challenges underscore that this field remains at an early stage of development [2,3,8,29].

Accordingly, future progress will depend on standardized large-animal studies integrating optimized cryoprotectant delivery, controlled rewarming technologies, post-thaw perfusion-based functional assessment, and clearly defined reproductive and vascular endpoints. Only through such rigorous preclinical validation can uterine cryopreservation be responsibly advanced toward broader translational relevance, whether as a model for organ banking research or as a potential component of future reproductive and transplant medicine.

## Conclusions

Available preclinical evidence indicates that uterine cryopreservation, particularly in porcine models, remains an experimental, hypothesis-generating approach to whole-organ preservation rather than a clinically established strategy. While controlled slow-freezing protocols can preserve uterine architecture and partial post-thaw contractility, functional recovery is incomplete, and no reproductive outcomes have been demonstrated following cryopreserved uterine transplantation. Evidence for whole-uterus cryopreservation is limited largely to slow-freezing techniques, with persistent technical challenges related to organ size, vascular complexity, cryoprotectant toxicity, and thermal heterogeneity during rewarming. Accordingly, the uterus represents an ethically appropriate preclinical model for refining preservation technologies, and further standardized large-animal studies with functional and reproductive endpoints are required before clinical application or organ banking can be responsibly considered.
